# Validation of GeneXpert testing for SARS-CoV-2 RNA in eight hospital laboratories in Oman

**DOI:** 10.11604/pamj.2021.40.2.27224

**Published:** 2021-09-01

**Authors:** Nawal Al-Kindi, Intisar Al-Shukri, Azza Al-Rashdi, Nada Al-Siyabi, Samira Al Mahruqi, Hanan Al-Kindi, Amina Al-Jardani

**Affiliations:** 1 Medical Microbiology, Khoula Hospital, Muscat, Oman,; 2 Central Public Health Laboratory, Darsait, Oman,; 3 Medical Microbiology, Al-Nahdha Hospital, Muscat, Oman

**Keywords:** COVID-19, SARS-CoV-2, comparison, coronavirus, RT-PCR

## Abstract

In response to the current COVID-19 pandemic, numerous commercial assays have been developed for the detection of SARS-CoV-2 for use in the clinical diagnostic laboratories. To date, there is limited comparison of testing methods performed in different hospital laboratory sites. The aims of the study were to evaluate the analytical performance of Cepheid Xpert Xpress SARS-CoV-2 when compared to RT-PCR. This is a cross-sectional study. A total of 155 nasopharyngeal swabs were taken in duplicate from patients presenting with suspected COVID-19 to 8 hospitals in Oman. One swab was tested by the hospital laboratory and the duplicate swab was sent to the national Central Public Health Laboratory (CPHL) for testing. We compared the analytical performance of the commercially available point of care Cepheid Xpert Xpress SARS-CoV-2 assay which was used in the 8 different hospitals with assays including Liferiver, Sansure, TIB MOL BIOL, Kingfisher and COBAS 6800 by Roche which were performed at the CPHL. Testing of the duplicate swabs revealed excellent agreement of results with the viral loads of Ct values ranging from 16-43 for the E gene, 18-44 for the N gene and 17-44 for the ORF gene using the Liferiver assay. The overall sample sensitivity and specificity of the Cepheid Xpert Xpress SARS-CoV-2 assay were both 100% and there was 100% agreement across specimens. We conclude that the rapid GeneXpert and RT-PCR kits assessed in this study may be used for routine diagnostic testing of COVID-19 patients by experienced clinical microbiology diagnostic laboratories. Our results highlight the importance of rapid molecular testing at different sites within a country in a public health emergency.

## Introduction

Since the first case reported on December 31, 2019 in Wuhan City, Hubei Province, People's Republic of China [1], COVID-19 disease caused by SARS-CoV-2 virus continued to evolve and spread throughout the world to involve majority, if not all, the countries. The COVID-19 pandemic has had a major impact on clinical microbiology laboratories since its start, including pre-analytical, analytical and post-analytical phases of testing [2]. In several settings, it has been stated that the preferred testing method for SARS-CoV-2 virus is the real-time reverse transcriptase-PCR test [3-5]. Various assays have been developed including different gene targets e.g., N, E, RdRp and ORF with different international recommendations of which of these targets to use [4, 6].

In addition to real-time reverse transcriptase-PCR and in response to the COVID-19 pandemic, multiple real time PCR (RT-PCR) assays with less labour-intensive work and shorter turnaround time have been developed. Among these, Cepheid Xpert Xpress SARS-CoV-2 was one of those that received authorization for emergency use from the US Food and Drug Administration [7]. The targets in the assay are the viral envelope E gene and the nucleocapsid N2 gene. However, there were major limitations to its use, such as low throughput and high cost [8]. In Oman, at the beginning of the SARS-CoV2 pandemic, there were only two major laboratories that could perform molecular diagnosis of the infection. The CPHL served the whole country which led to the addition of transport issues, to prolonged turnaround times. In such settings, the GeneXpert test represents an excellent option for rapid diagnosis, in less than an hour, of critically ill patients in hospitals that can be distributed to serve different sites. In fact, only limited studies have been conducted to evaluate the performance of such tests [8-11].

This study aims to evaluate the analytical performance of Cepheid Xpert Xpress SARS-CoV-2 in comparison with RT-PCR including sensitivity, specificity, agreement and correlation between the new test and the gold standered RT PCR.

## Methods

**Study design and setting:** this is a cross-sectional study where nasopharyngeal swabs were collected from patients who met the case definition of patients suspected to have COVID-19 according to national guidelines [1, 12] in 8 different regional hospitals (Sultan Qaboos, Sohar, Nizwa, Ibri, Alnahdha, Khasab, Rustaq and Khoula Hospital). These hospitals are regional hospitals representing all the governorate throughout the country and serve the Omanis population.

**Study population:** a total of 155 replacement swabs (approximately between 10-20 swabs from each site, which is considered adequate number according to international bodies for validation) were collected in the regional hospitals during the period of equipment validation between March and June 2020 according to each site testing capacity.

**Sampling technique:** one specimen of each duplicate was processed on the GeneXpert (Xpert, Cepheid, Sunnvale, CA, USA) and the other specimen was sent to the CPHL for confirmation. All details of the results, including the CT values, were recorded on the request forms. All samples were extracted using the Liferiver extraction kit according to manufacturer´s recommendations. Most of the samples were then tested using commercial assays. The Liferiver assay detects three genes: ORF1 ab, N and E. A few samples (4 and 5 respectively) were tested using the Cobas® SARS-CoV-2 assay on the Cobas 6800 system (Roche) and Novel Coronavirus (2019-nCoV) Nucleic Acid Diagnostic Kit, CE-IVD, FDA-EUA (Sansure, Biotech).

**Data analysis:** statistical analysis between the results obtained in the duplicate swabs was performed using the Mann-Whitney U test for independent samples. The statistical tests were two tailed with a significance level of 0.05. MedCalc Statistical Software version 19.0.5 (MedCalc Software bvba, Ostend, Belgium) was used for statistical analysis. Standard statistical measures for test performance were assessed to determine the sensitivity (the proportion of samples that were correctly identified as positive), specificity (the proportion of negatives that are correctly identified), positive predictive value (the probability that a positive test result indicates that the patient has infection) and the negative predictive value (the probability that a negative test result indicates that the patient does not have infection).

Ethical approval: not applicable as the study was done in the national central laboratory where the gold slandered testing was done.

## Results

A total of 155 nasopharyngeal swabs were taken in duplicate from patients presenting with suspected COVID-19 to 8 hospitals in Oman; Sultan Qaboos Hospital (n=22), Sohar Hospital (n=24), Nizwa Hospital (n=18), Ibri Hospital (n=21), Al Nahdha Hospital (n=19), Khasab Hospital (n=22), Rustaq Hospital (n=9) and Khowla Hospital (n=20). One swab was tested by the hospital laboratory and the duplicate swab was sent to the Central Public Health Laboratory (CPHL), for testing. The various hospital laboratories used the Cepheid Xpert Xpress SARS-CoV-2 assay, whilst the Liferiver Novel Coronavirus (2019-nCoV) Real Time RT-PCR Kit and other assay kits were used at CPHL.

Of the 155 swabs tested in parallel, 39 (25.16%) had detectable SARS-CoV-2 viral loads with Ct values ranging from 15-43 for the E gene and 17-44 for the N gene using the Xpert Xpress SARS-CoV-2 assay in the 8 hospital laboratories (Table 1). Testing of the duplicate swabs revealed excellent agreement of results with the viral loads of Ct values ranging from 16-43 for the E gene, 18-44 for the N gene and 17-44 for the ORF gene using the Liferiver assay.

**Table 1:** comparison of the Xpert Xpress and Liferiver RT-PCR assays for SARS-CoV-2 detection

Sample ID	Xpert Xpress		Liferiver		
	E gene	N gene	E gene	Ngene	ORF gene
1	44	44.1	36.1	35.55	34.38
2	28.8	31.5	33.19	34.57	35.12
3	36.4	38.7	35.38	44	38.41
4	29.15	30.1	29.15	27.83	33.37
5	21.3	23.8	21.4	23.86	24.42
6	26.3	28.1	30.58	32.11	28.87
7	24.2	26.6	16.13	18.29	17.97
8	21.7	24.1	nt	nt	nt
9	18.3	20.4	18.6	19.9	20.6
10	15.1	17.2	nt	nt	nt
11	27.8	30.2	nt	nt	nt
12	31.6	34.8	32.48	37.73	33.72
13	23.4	25.6	23.82	35.59	25.21
14	24.6	26.6	nt	nt	nt
15	44	42	44		
16	35.6	37.7	33.93	37.33	36.55
17	43.2	42.2	44	44	38.24
18	44	39.5	36.83	44	44
19	33.4	36.5	35.5	37.53	38.01
20	28.4	31.7	30.9	34.4	32.4
21	16.9	19.2	18.6	21.1	20.2
22	30.2	33.3	31.4	40.3	33.8
23	22.1	24.4	23.48	25.07	24.52
24	35.6	39.4	34.63	41.61	35.45
25	32.4	35.2	nt	nt	nt
26	33.7	36.8	34.47	36.71	35.16
27	29.6	31.9	34.5	31.8	32.3
28	38	40	nt	nt	nt
29	34	33	nt	nt	nt
30	29	32	19.6	22.24	21.7
31	18	20	18.47	20.58	20.73
32	16	18	18.6	33.45	32.28
33	29	33	31	33.45	32.28
34	27	30	29.75	31.04	30.91
35	30.3	32.4	nt	Nt	nt
36	19.2	21.9	nt	Nt	nt
37	29.1	31.3	27.5	30.4	29.2
38	31	33.6	nt	Nt	nt
39	37.2	39.2	nt	Nt	Nt

Results are shown as Ct values. nt denotes not tested.

**Figure 1:**
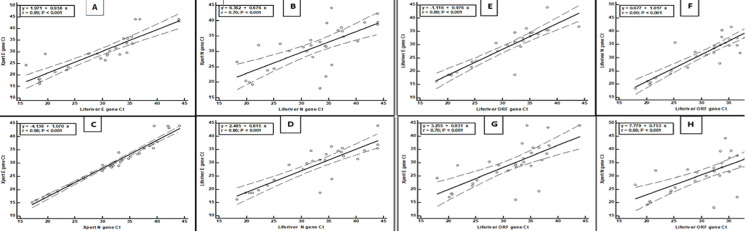
scatter diagram showing the regression line of Ct values between the Xpert Xpress and Liferiver SARS-CoV-2 assays and the target viral genes for the positive samples; A) Xpert E gene Ct vs Liferiver E gene Ct, B) Xpert N gene Ct vs Liferiver N gene Ct, C) Xpert E gene Ct vs Xpert N gene Ct, D) Liferiver E gene Ct vs Liferiver N gene Ct, E) Liferiver E gene Ct vs Liferiver ORF gene Ct, F) Liferiver N gene Ct vs Liferiver N gene Ct, G) Xpert E gene Ct vs Liferiver ORF gene Ct, H) Xpert N gene Ct vs Liferiver ORF gene Ct; The regression equation, correlation coefficient (r) and p values are shown; The solid line represents the regression and the broken lines represent the 95% confidence levels

The overall test performance of the GeneXpert assay compared to the Liferiver RT-PCR showed that sensitivity and specificity were both 100% (Table 2). As shown in Figure 1A-H, the high coefficients of determinations were observed for Ct values between the assays of the target viral genes for the 39 positive samples and were as follows; A) Xpert E gene Ct vs Liferiver E gene Ct, r=0.89, p<0.001; B) Xpert N gene Ct vs Liferiver N gene Ct, r=0.70, p<0.001; C) Xpert E gene Ct vs Xpert N gene Ct, r=0.98, p<0.001; D) Liferiver E gene Ct vs Liferiver N gene Ct, r=0.86, p<0.001; E) Liferiver E gene Ct vs Liferiver ORF gene Ct, r=0.89, p<0.001; F) Liferiver N gene Ct vs Liferiver ORF gene Ct, r=0.90, p<0.001; G) Xpert E gene Ct vs Liferiver ORF gene Ct, r=0.70, p<0.001; H) Xpert N gene Ct vs Liferiver ORF gene Ct, r=0.69, p<0.001. Of interest, several other commercial SARS-CoV-2 RT-PCR assays including Sansure, Kingfisher and Cobas 6800 from Roche were used at CPHL with 100% concordance (data not shown).

**Table 2:** diagnostic test performance measurement and statistical analysis of the GeneXpert assay compared to the Liferiver RT-PCR for the detection of SARS-CoV-2 RNA

		RT-PCR		
		Detected	Not Detected	Total
**GeneXpert**	Detected	39	0	39
	Not Detected	0	116	116
**Total**		39	116	155
Sensitivity:39÷ (39+0) =100%				
Specificity:116÷ (116+0) =100%				
Positive predictive value(PPV):39÷ (39+0) =100%				
Negative predictive value(NPV):116÷ (116+0) =100%				

## Discussion

In this study, we compared the analytical performance of the commercially available Cepheid Xpert Xpress SARS-CoV-2 assay which was used in 8 different hospitals with the Liferiver and other RT PCR assays performed at the Central Public Health Laboratory in Oman on a total of 155 nasopharyngeal swabs. Molecular assays target different genes in the SARS-CoV-2 genome, mainly the nucleocapsid (N), envelope (E), non-structural protein (Nsp)2, and open reading frame ORF1/2. The N1 and N2 targets within the N gene were recommended by the CDC [13, 14] whereas the WHO recommends an initial screening with the E gene followed by confirmation with the RNA dependent RNA polymerase (RdRp) [4].

Our findings and other published data illustrate that the assay performance for SARS-CoV-2 detection is not dictated by the selected gene. However, a recent report concluded higher sensitivity of primers that target the N2 or the E genes [15]. In agreement with our findings, it has been reported that the targeting of two genes appears to enhance the assays´ sensitivities and could also reduce the risk of sensitivity reduction associated with genomic polymorphism mutations [16].

As previously reported, the potential limitations in our study include differences in the sample input volume for each assay, differences in amount of extracted RNA included in the RT-PCR reaction, as well as differences in the extraction efficiency between assays [17]. Although these confounding factors cannot be accurately accounted for, our findings demonstrate 100% agreement in the detection of SARS-CoV-2 by the assays.

## Conclusion

With the critical future need for the use of molecular SARS-CoV-2 diagnostics not only for diagnosis, but also for asymptomatic large-scale screening, it is essential that consistency between different hospital laboratories is confirmed to inform decisions related to contact isolation and measures that are essential to mitigate the current pandemic in Oman or any other country. This comparison study showed good correlation and analytical performance between the commercially available Cepheid Xpert Xpress SARS-CoV-2 assay used in the regional hospitals compared with the Liferiver and other RT PCR assays used in the national central laboratories.

### What is known about this topic

- The Xpert Xpress SARS-CoV-2 test is a rapid, real-time RT-PCR test intended for the qualitative detection of nucleic acid from the SARS-CoV-2 in upper respiratory specimens (such as nasopharyngeal, oropharyngeal or nasal swabs, wash or aspirate) collected from individuals suspected of COVID-19;
- Negative results do not preclude SARS-CoV-2 infection and should not be used as the sole basis for treatment or other patient management decisions;
- The Xpert Xpress SARS-CoV-2 test is only for use under the Food and Drug Administration´s Emergency Use Authorization.

### What this study adds

- There is a good correlation and analytical performance between the commercially available Cepheid Xpert Xpress SARS-CoV-2 assay with the Liferiver and other RT PCR assays.
- In agreement with our findings, it has been reported that the targeting of two genes appears to enhance the assays´ sensitivities and could also reduce the risk of sensitivity reduction associated with genomic polymorphism mutations reported widely.
- Our findings demonstrate 100% agreement in the detection of SARS-CoV-2 by the assays compared (GeneXpert vs RT PCR).
